# Conditioned Medium – Is it an Undervalued Lab Waste with the Potential for Osteoarthritis Management?

**DOI:** 10.1007/s12015-023-10517-1

**Published:** 2023-02-15

**Authors:** Monika A. Rosochowicz, Michał S. Lach, Magdalena Richter, Wiktoria M. Suchorska, Tomasz Trzeciak

**Affiliations:** 1 Department of Orthopedics and Traumatology, Poznan University of Medical Sciences, 28 Czerwca 1956r. 135/147 Street, 61-545 Poznan, Poland; 2 Radiobiology Laboratory, Greater Poland Cancer Centre, Garbary 15 Street, 61-866 Poznan, Poland; 3 Department of Electroradiology, Poznan University of Medical Sciences, Garbary 15 Street, 61-866 Poznan, Poland

**Keywords:** OA, Osteoarthritis, MSCs, CM, Stromal cells, Chondrocytes, Exosomes

## Abstract

**Background:**

The approaches currently used in osteoarthritis (OA) are mainly short-term solutions with unsatisfactory outcomes. Cell-based therapies are still controversial (in terms of the sources of cells and the results) and require strict culture protocol, quality control, and may have side-effects. A distinct population of stromal cells has an interesting secretome composition that is underrated and commonly ends up as biological waste. Their unique properties could be used to improve the existing techniques due to protective and anti-ageing properties.

**Scope of Review:**

In this review, we seek to outline the advantages of the use of conditioned media (CM) and exosomes, which render them superior to other cell-based methods, and to summarise current information on the composition of CM and their effect on chondrocytes.

**Major Conclusions:**

CM are obtainable from a variety of mesenchymal stromal cell (MSC) sources, such as adipose tissue, bone marrow and umbilical cord, which is significant to their composition. The components present in CMs include proteins, cytokines, growth factors, chemokines, lipids and ncRNA with a variety of functions. In most in vitro and in vivo studies CM from MSCs had a beneficial effect in enhance processes associated with chondrocyte OA pathomechanism.

**General Significance:**

This review summarises the information available in the literature on the function of components most commonly detected in MSC-conditioned media, as well as the effect of CM on OA chondrocytes in in vitro culture. It also highlights the need to standardise protocols for obtaining CM, and to conduct clinical trials to transfer the effects obtained in vitro to human subjects.

**Graphical Abstract:**

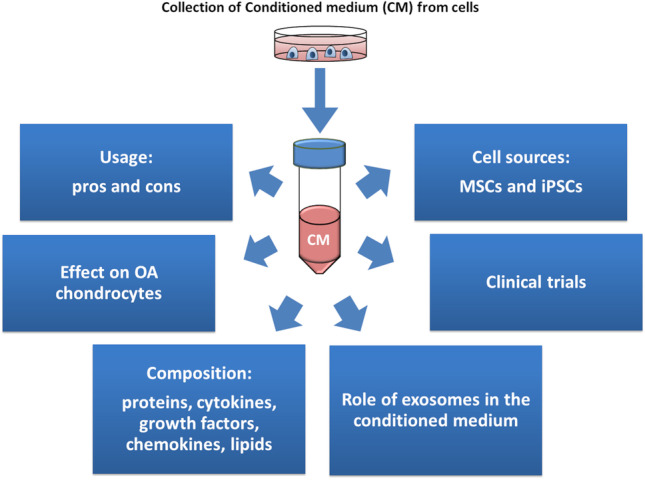

## Background

Hyaline cartilage is a subtype of connective tissue that provides a smooth surface reducing friction and resistance to compressive forces during movement [1–3]. Its function is made possible due to a dense extracellular matrix (ECM) [1, 2, 4, 5], consisting of a network of collagen fibres, proteoglycans, and non-collagenous proteins [1, 4, 6]. Cartilage is basically composed of a single cell type –chondrocytes [1, 4], which once the organism has reached skeletal maturity, are unable to divide and maintain the quiescent state [6]. These cells are responsible for synthesising ECM components and proteolytic enzymes, that remodel surrounding tissue [4, 6]. The histological architecture of the cartilage also includes neither blood, lymphatic vessels nor neural tissue, which in general provides an ease of movement. The features of this tissue consequently preclude the regeneration of damaged tissue, which may lead to the development of osteoarthritis [7].

OA is a chronic degenerative disease, the development of which is affected by several factors, such as obesity, genetic predispositions, age and extensive stress on the joints [4, 8, 9]. This damages the dense ECM and affects the secretome of the local microenvironment, disrupting the balance between catabolic and anabolic actions in the metabolism of the chondrocytes. The primary causes of the idiopathic disease are yet to be fully explained, but it is thought to be closely related to the inflammatory process. Unlike any other arthritis, however, it is directly related to a severe inflammatory response with an autoimmune background [10]. The interleukin 1β (IL-1β) and tumour necrosis factor α (TNFα) are the most prominent cytokines involved in the progression of OA and the most commonly used during in vitro modelling [4, 5, 9]. The secretion of these inflammatory mediators leads to the release of even more chemokines and pro-inflammatory cytokines, as well as ECM-degrading enzymes: matrix metalloproteinases (MMPs) and aggrecanases [4, 9].

Among the ways of treating OA, we may distinguish methods consisting mainly in fighting the symptoms of the disease, such as the use of painkillers or joint injections, e.g. with hyaluronic acid (HA), corticosteroids or platelet-rich plasma (PRP) [3, 5, 11–13]. In addition to symptomatic treatment, there is also surgical management, which involves: microfracture, the use of cells and tissue engineering (autologous chondrocyte implantation (ACI) or matrix-induced autologous implantation (MACI)), osteochondral autograft transplantation and alloplasty, the most invasive [1, 5, 11, 14]. Comparisons of these methods, together with their pros and cons have been already described in a number of reputable studies [5, 14, 15]. The main drawbacks of these procedures tend to be the formation of fibrocartilage, the lifespan of the implants and the invasiveness of these procedures. New sources of cells and materials for tissue engineering purposes are therefore necessary, as are alternative methods for preventing the development of OA or significantly retarding it [1, 11, 15, 16]. This would offer a better quality of life and delay extensive surgical interventions.

Cell engineering has mainly been applied in ACI and MACI procedures [3–5, 13]. Initially, it involves collecting chondrocytes from the patient, cultivating them through cell culture and retransplanting them into the site of the defect [4]. The main advantage of cell-based therapies is that the cellular material needed can be taken from various sources, which is extremely hopeful for patients with degenerative diseases, elderly patients and those with genetic and metabolic dysfunctions [3, 15]. Neither do cell-based methods require complex surgery, reducing invasiveness [1, 15, 17]. Among the MSCs that may be used in therapy a variety may be distinguished, but not exclusively osteoblasts, chondrocytes, adipocytes, astrocytes and cardiomyocytes, and they exhibit anti-inflammatory outcomes. They may therefore make it possible to reconstruct the joint and, through immunomodulation, reduce the locally induced immune response [1, 4, 11, 18]. The availability of autologous material for the treatment minimises the risk of patients rejecting it [1, 4]. The efficiency of cell therapy is to a great extent determined by the donor’s health and age, as well as the properties and viability of the cells collected [15]. One of the risks associated with cellular-based therapies is insufficient integration of the cells at the site of the implantation, which significantly reduces the efficacy of the treatment [19–21]. The cost of cell therapy, adequately qualified staff and suitable facility core are other limitations on its everyday clinical use.

These limitations have led to the search for new solutions involving various cell populations in the management of OA. The use of MSCs has emerged as a promising source, but it has failed to eliminate most of the drawbacks. During the 1970s and 1980s it was observed that MSCs owe their effect largely to paracrine activity, which has led to research on CM as a cellular substitute for regenerative purposes [22]. Secreted factors are thought to act even in the absence of cells leading to tissue repair [23]. Up to 80% of the regenerative potential initially attributed to transplanted cells also belongs to paracrine-secreted cellular factors [24].

This review will summarise recent data on the suitability of conditioned media from different cellular sources for cartilage regeneration purposes.

## New Promising Treatment Approaches: The Conditioned Medium

The term 'conditioned medium' refers to the liquid phase of a cell culture environment enriched with the secretome of cultivated tissue [22]. Its composition is affected by various factors in the microenvironment of the cell culture, including the inhibition of cell contact, cell aggregation, cell growth and differentiation ability, and physical and chemical parameters [22].

Therapies based on a combination of CM with an appropriate population of cells could be an alternative or might improve existing procedures. Among the advantages that may determine the superiority of CM and the agents they contain over cell-based therapies is immunocompatibility. The exclusion of cells obviates the selection of donors and recipients in therapy [21, 23].

Additionally, cells prepared for the therapy die in culture or during delivery to the site of the defect, whilst, CM contains no live material and therefore entails no such inconvenience [24]. CM is easily manufactured, packaged, frozen and transported [23]. Laboratory sterility is only needed at the stage of CM production, while media application may occur under non-sterile conditions [21, 23]. In addition, CM is produced under controlled laboratory conditions, to enable inspection of the composition [24]. CM may therefore easily be applied to regenerative therapies that require only ambulatory conditions. Time and cost could also be saved in therapies involving CM, as the method requires no waiting for the cells reach maturity and it is possible to obtain the medium several times from cells that have been seeded only once [25].

Like any method, the use of CM in regenerative medicine as a new field carries many unknowns. One such unknown is the lack of common recommendations and standards for bioprocessing and quality control of CM-based therapies, resulting in a wide variation and a composition depending on the method and the time of culture [26]. Possible hypersensitivity reactions related to the components of the base medium administered to the cells to obtain the conditioned medium may also be a risk. The long-term effect of their use is not well known, due to the low number of clinical trials in this area. The detailed list of advantages and disadvantages is summarised in Table 1.

**Table 1:** Advantages and disadvantages of conditioned media *versus* cell transplantation [21, 23–25, 27]

Advantages	Disadvantages
+ reduced risk of graft-versus-host disease; + fast and cheap production: multiple CM from one culture; + easy storage and transport; + application procedure requires no sterile conditions: enhanced convenience of usage; + less invasive administration: requires no surgery; + prolonged shelf-life due to the absence of cells; + ready-to-use biological products as pharmaceuticals for regenerative medicine; + lower risk of tumourigenesis due to cell-free composition	- incomplete knowledge of composition and mechanism of factors; - the necessity to optimise the production in order to standardise the composition of CM; - high variability depending on cell culture, type, passage number; - different levels of factors depending on the culture condition and CM processing methods; - few clinical trials; - allergenic reactions to base medium compositions

The issues mentioned above could be rendered relatively meaningless against the benefits from their application. Gunawardena et al. emphasise that a conditioned medium could be used effectively in managing a number of conditions, so it is necessary to identify the factors responsible for treating or inhibiting OA. A few aspects must, however, be established: the specific knowledge regarding CM components, dosage and delivery method to gain a favourable outcome [21, 28]. It is crucial to select a suitable source of cells secreting specific paracrine factors.

In vitro studies allow for the examination of individual patients' responses to the administration of the CM, so that the semi-retention period of the medium factors in the body may be partially determined. The efficacy of CM-mediated treatment may also be greatly enhanced by modifying the medium composition with patient-specific factors at the manufacturing level, which will enable the development of safe and efficacious, personalised therapies for many conditions [21].

## Conditioned Media: Cell Sources

The elements essential in order to produce the CM are cells, most with stromal cells abilities, since these cells have been proven to secrete a variety of cytokines, growth factors and exosomes, responsible for therapeutic effect and tissue regeneration [29–32]. The most commonly used source across studies are MSCs, due to their capacity for self-renewal and their multipotent nature [25]. They migrate to the site of the damaged tissue and differentiate into the desired cell types, rendering them essential in tissue repair [33]. Another equally interesting aspect of MSC-mediated tissue repair is the fact that these cells secrete several molecules that activate and mobilise cells at the site of the damage, contributing to the modulation of the inflammatory process, which is also thought to accelerate the healing of tissue [34].

In this review the abbreviation MSC stands for 'mesenchymal stromal cells' and refers to work published by Caplan, who has repeatedly emphasised that the term 'stem cells' is misused for what MSCs represent and maintains that they should be referred to as mesenchymal stromal cells. This may confuse clinicians, researchers and patients [35, 36]. The International Society for Cell and Gene Therapy (ISCT®) has defined the minimum criteria cells must fulfil to qualify as MSCs. Firstly, MSCs must be plastic-adherent in in vitro culture. Secondly, they must be characterised as expressing CD105, CD73 and CD90, but lack expression of CD45, CD34, CD14, CD11b, CD79α, CD19 and human leukocyte antigen-DR surface molecules. Lastly, they must differentiate in vitro into osteoblasts, adipocytes and chondrocytes [37]. Caplan emphasises that mesenchymal stem cells are often used for cell populations that fail to fulfil the above characteristics, leading to a misunderstanding and misinterpretation in studies [36]. This paper therefore employs the term 'stromal cells' for MSCs for a more precise explanation of the topic.

Induced mesenchymal stromal cells (iMSCs) have been extensively used in cartilage regeneration therapies because their properties allow multilineage differentiation. Additionally, the MSC secretome modulates the anti-inflammatory and immunosuppressive properties of the microenvironment and protect cartilage from further damage [38]. The differentiation of MSCs into chondrocytes at the site of injury may also lead to a reduction in prostaglandin concentration in synovial fluid, which could reduce the progression of OA [38]. In general, MSCs also display the ability to inhibit the proliferation and the activation of immune cells (T cells, B-cells, NK cells (natural killer cells), and DC (dendritic cells)) [39].

**Fig. 1:**
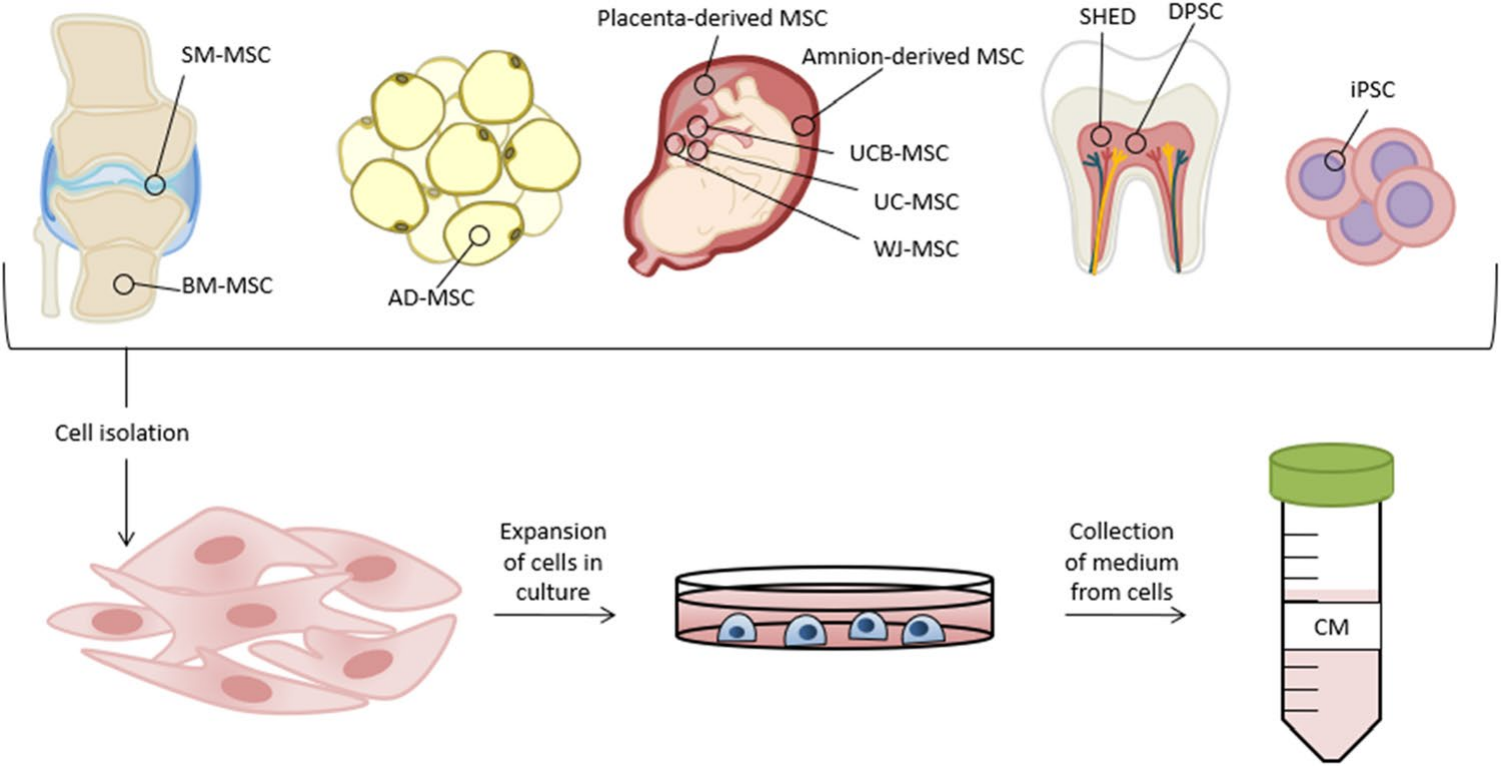
The commonly used cell sources and obtaining the conditioned medium. CM for cartilage regeneration therapies may be obtained from stromal and stem cell populations such as BM-MSCs, SM-MSCs, AD-MSCs, placenta-derived-MSCs, amnion-derived-MSCs, UC-MSCs, UCB-MSCs, WJ-MSCs, DPSCs, SHED and iPSC (induced pluripotent stem cells). Isolated homogenic populations of cells are expanded in in vitro culture. After a reasonable period of time or when adequate cell confluence is achieved CM might be collected, precleared and stored for the future use. circRNA, circular RNA; CCL2, C–C motif chemokine ligand 2; DKK-1, dickkopf 1; ECM, extracellular matrix; FGF, fibroblast growth factor; HGF, hepatocyte growth factor; HIPK3, homeodomain interacting protein kinase 3; IFN-γ, interferon γ; IL-6 /-10, interleukin 6 /10; LIF, leukemia inhibitory factor; lncKLF3-AS1, KLF Transcription Factor 3 Antisense RNA 1; lncRNA, long-non coding RNA; LYRM4, LYR Motif-Containing Protein 4; miR, micro RNA; MMPs, matrix metalloproteinases; ncRNA, non-coding RNA; PDGF, platelet derived growth factor; PGE2, prostaglandin-E2; SDF-1, stromal cell-derived factor 1; SEA, N-stearoylethanolamide; SERPINE, serine proteinase inhibitor; TGF-β, transforming growth factor β; TIMPs, tissue inhibitor of metalloproteinases; VEGF, vascular endothelial growth factor; VIM, vimentin

The most common sources of stromal cells are bone marrow-MSCs (BM-MSCs), synovial membrane-MSCs (SM-MSCs), adipose tissue-derived-MSCs (AD-MSCs), placenta-derived-MSCs, amnion-derived-MSCs, umbilical cord-MSCs (UC-MSCs), umbilical cord blood-MSCs (UCB-MSCs), Wharton's jelly-MSCs (WJ-MSCs), dental pulp stromal cells (DPSCs), and also human exfoliated deciduous teeth stromal cells (SHED), shown in Fig. 1.

The most commonly used and best-characterised stromal cells in regenerative therapies are BM-MSC and AD-MSC. They differ in their origin and their properties, i.e. in the secreted cytokines. The lack of a defined protocol for obtaining CM means there is a great variability among the CMs discussed in the studies [40]. This was emphasised in the study by Alexandrushkina et al., who investigated the secretome of MSC cultured in monolayers and cell sheets (CS). They demonstrated that CS contain more factors that are involved in chemotaxis and the maturation of blood vessels [41]. Zisa et al. also demonstrated that the number of secreted trophic factors depends on the origin of the cells and showed that human BM-MSC secretes less vascular endothelial growth factor (VEGF) than porcine BM-MSCs [42]. The most common sources of CM are AD-MSC [34, 41, 43], BM-MSC [44–46], UC-MSC or WJ-MSC [34, 44, 47] and SHED [30, 31, 44]. The effect of the conditioned medium will also vary depending on the type of cells to which the CM is administrated and the time, method and conditions under which the test cells are cultured. Each cell type was found to differ from the others in the terms of the secreted factors: AD-MSCs secreted collagen I, II and III at the highest levels; WJ-MSCs secreted small amounts of ECM components; BM-MSCs secreted higher amounts of heparin sulphate than the other cells [34]. Cells derived from adipose tissue also secrete higher amounts of pro-angiogenic factors, including VEGF, hepatocyte growth factor (HGF), fibroblast growth factor (FBF), platelet-derived growth factor (PDGF) and angiopoietin-1 (Ang-1) and angiopoietin-2 [48]. AD-MSCs, however, secrete the highest amount of matrix metalloproteinases (MMP-1, MMP-3, MMP-13) compared to WJ-MSCs and BM-MSCs [34].

Their origin means that WJ-MSCs have a greater regenerative capacity and exhibit a better proliferative potential, but become problematic when the patients cryopreserve no umbilical cord cells [34]. Wharton's jelly mesenchymal stromal cells are an attractive source of cells for therapy, due not only to their high proliferative capacity, non-controversial nature and ability to differentiate into three germ lineages, but also because of their prolonged stemness trait and non-cancerous character [49].

Researchers also indicate the regenerative potential of DPSCs. Kichenbrand et al. emphasised that DPSCs represent an attractive option for obtaining the MSCs. A dental pulp source provides a large number of cells. Isolation may be performed by enzymatic and mechanical methods from adult or baby teeth during ordinary dental procedures. DPSCs display a higher proliferation and better ability to induce mineralisation than BM-MSCs, making them a promising cell source for OA therapy. These cells can also be combined with scaffolds to achieve even better anti-inflammatory and immunomodulatory effects in regenerative therapies [50]. Nosrat et al. also emphasise that DPSCs produce neurotrophic factors in culture, promoting neuron survival. Their neuroregenerative properties were also demonstrated when transplanted into the spinal cord [51]. This transplantation led to an increased number of rescued motoneurons, indicating the significant role for factors secreted by DPSCs in cell proliferation [51]. In contrast, Wang et al. demonstrated that SHEDs, also derived from dental pulp, exhibit a higher proliferation rate, better differentiation and mineralisation capacity after transplantation than DPSCs [52]. Compared to DPSCs, SHEDs are characterised by a higher expression of MMP-1 and MMP-2, which may discourage the use of these cells in the treatment of articular cartilage. Factors showing elevated expression in SHED cells also include tissue inhibitor of metalloproteinases 1 (TIMP-1), tissue inhibitor of metalloproteinases 2 (TIMP-2) and interleukin 6 (IL-6), which promote the regeneration of cartilage tissue [52].

iPSCs are another promising source for regenerative therapies. Due to their foetal-like tissue nature, it is suspected that they may act more efficaciously in regenerative therapies than MSCs. iPS cells are derived from adult somatic cells and then genetically reprogrammed into embryonic stem-like (ES-like) cells. Because they are obtained as in a laboratory rather than isolated from the blastocyst’s inner mass they entail no ethical issues [53, 54]. Due to the ability of iPS cells to differentiate into cartilage tissue cells, they may be used in cell-based OA therapies such as ACI and MACI procedures [55–57]. iPS cells may also be used to design disease models, e.g. for OA, in order to study pathological processes during the disease more precisely [56]. Using these cells in therapies may, however, carry the risk of teratoma formation [55]. Tissues generated from iPS cells will be nearly identical to donor cells, making them almost ideal for regenerative medicine [53] if only precise and effective protocols are developed for differentiating iPSCs into chondrocytes.

The CM above iPS cells is yet to be explored for use in the context of OA, but it has been shown to enhance proliferation, migration and maintenance of morphology in bovine corneal endothelial cells (B-CECs) [58]. The action of iPSC-CM on endothelial cells is thought to involve phosphorylation of the Akt signalling pathway and regulation of the PI3-kinase pathway, as well as leading to an increase in the expression of activin-A [58]. In another study Hsieh et al. demonstrated that iPSC-CM protects against tumour-induced immunosuppression by increasing the activity of host NK cells [59]. Su et al. found that CM of iPSCs reduced neutrophil recruitment associated with the pathogenesis of acute lung injury by inhibiting their endothelial migration and promoting endogenous leukaemia inhibitory factor (LIF), as well as reducing the endothelial leakage of immune cells [60]. Researchers also highlight that the medium reduces NF-κB activity and neutrophil chemotaxis [60]. Zhou et al. demonstrated that the application of iPSC-CM leads to improved lung structure in mice with pulmonary fibrosis and promotes the repair of damaged tissue by inhibiting the Transforming growth factor β1/Suppressor of Mothers Against Decapentaplegic (TGF-B1/Smad) signalling pathway [61]. This confirms the protective effect of CM on lung tissue and its involvement in the prevention of pulmonary fibrosis. The above-mentioned studies demonstrate the enormous potential of iPSC-CM in the treatment of many diseases, especially in the context of regenerative medicine.

## Composition of Conditioned Media

The idea of testing the composition of CM emerged from the unexplained association between efficacious MSC engraftment and improved functionality at the site of implantation [35, 62]. Observations suggested that the process might involve the paracrine properties of these cells [21, 23, 48]. The increasing interest in the secretome of MSCs has allowed the initial identification of several elements with which CM is enriched: proteins, lipids, nucleic acids and extracellular vesicles [40, 63]. Researchers have further distinguished a group of molecules that includes cytokines, chemokines, receptors, growth factors and inflammatory factors [64, 65]. The function of major CM components in chondrocyte biology is shown in Table 2. It is important to highlight that the compositions of presented CM are depend largely on the type of cells from which they are derived, the method and conditions of cell culture, the media used and the methods of purification.

**Table 2:** Function of major conditioned medium components in the context of the biology of chondrocytes

Component	Function	Ref	Proved presence
Proteins			
MMP-2	- degradation and remodelling of ECM; - involvement in inflammatory processes; - promotion of cell attachment; - promotion of proliferation and differentiation; - promotion of programmed cell death; - regulation of the destruction of cartilage by TGF-β activity; - recruitment of osteoblasts involved in tissue remodelling	[66–69]	[21, 43, 70, 71]
MMP-9	- degradation of ECM; - involvement in inflammatory processes; - recruitment of osteoclasts into hypertrophic zones; - formation of endochondral bone	[66, 68, 69]	[21, 43, 70]
MMP-13	- cleaving of collagen types I, II, III; - degradation of cellular matrix and non-cellular components	[72]	[30]
MMP-19	- degradation of aggrecan, COMP proteins, type IV collagen, type I gelatine, laminin, fibronectin and nidogen	[73, 74]	[75]
TIMP-1	- inhibition of fibrotic activity; - prevention of cartilage degradation; - inhibition of MMPs activity; - suppression of pain	[43, 71, 76–78]	[43, 70, 71, 76]
TIMP-2			[43, 70]
VIM	- promotion of chondrogenic differentiation of MPC; - regulation of the synthesis of the ECM; - enhancement of the expression of the cartilage markers; - maintenance of the chondrocytes phenotype	[79, 80]	[71, 81]
SERPINE1	- prevention of cartilage degradation; - tissue remodelling; - fibre formation; - inhibition of plasmin and cellular matrix metalloproteinases	[82]	[76, 81]
SERPINE2	- prevention of cartilage breakdown; - inhibition of MMP-13 expression	[83]	[81]
Cytokines, growth factors and chemokines			
TGF-β	- tissue development; - chondrocyte differentiation from early to final stages; - proliferation and differentiation; - maintenance of the chondrocytes; - inhibition of hypertrophy; - reduction of oxidative stress	[84, 85]	[30, 44, 64, 75, 86–88]
CCL2	- inflammatory response	[89]	[21, 76, 86, 90, 91]
VEGF	- proliferation and migration of endothelial progenitor cells; - chondrocyte survival; - endochondral ossification; - angiogenesis; - inflammatory process; - pain receptor sensitisation	[92–95]	[40, 44, 75, 86–88, 96]
HGF-1	- cell survival and proliferation; - cellular matrix metabolism; - inflammatory response; - osteochondral turnover; - bone remodelling; - regeneration of osteoarticular tissues	[97]	[21, 40, 44, 86, 87, 96]
LIF	- proteoglycans resorption in cartilage; - inflammatory and catabolic processes; - cell differentiation; - acute phase protein synthesis; - stimulation of calcium ion release in bone; - immune response; - cartilage degradation in arthritis	[98–101]	[81, 86]
FGF-2	- inhibition of aggrecan degradation; - TIMP-1 synthesis; - MMP-1 and MMP-3 synthesis; - cartilage protection; - inhibition of cartilage components degradation	[102, 103]	[40, 44, 96]
PDGF	- chemotaxis; - angiogenesis; - cell mitosis; - inhibition of inflammatory processes; - anti-apoptotic; - increase in the level of type II collagen; - reduction in the level of collagen X	[104, 105]	[21, 44, 64, 75, 87]
IFN-γ	- communication between immune cells; - activation of defence mechanisms; - polarisation of M1 macrophages; - ROS production; - production of pro-inflammatory cytokines; - bone resorption	[106, 107]	[44, 64, 87, 88]
DKK-1	- bone remodelling; - joint degradation; - chondrocyte ageing	[108–110]	[64, 76, 91]
IL6	- induction of the expression of ICAM-1; - downregulation of type II collagen expression; - pro-inflammatory influence; - production of B lymphocytes; - production of antibodies; - bone homeostasis; - differentiation of osteoclast; - pathogenesis of vascular inflammation; - the balance between the modulation of MMPs and TIMPs	[98, 111–114]	[44, 46, 75, 76, 88, 90]
IL10	- inhibition of inflammation markers; - upregulation of IL-1Ra and TIMP; - chondroprotection; - reduction of apoptosis of cartilage tissue cells	[111, 115, 116]	[44, 87, 88]
SDF-1	- induction of chondrocyte apoptosis; - increase in the inflammatory process in OA; - increase in the expression of IL-1, TNFα and MMPs; - reduction in the expression of COL2 and ACAN	[117–120]	[21, 44, 64]
Lipids			
PGE2	- stimulation of IL-10 production; - reduction of apoptosis in hepatocytes; - downregulation of the expression of SERPINE1/ PAI-1; - reduction in the expression of ACAN; - secretion of plasmin activator; - promotion of extracellular matrix degradation	[64, 82, 86, 121]	[40, 64, 86]
SEA	- downregulation of the expression of pro-inflammatory factors	[64, 122]	[64]

*ACAN* aggrecan, *CCL2* C–C motif chemokine ligand 2, *COL2* collagen type II, *COMP* cartilage oligomeric matrix protein, *DKK-1* dickkopf 1, *ECM* extracellular matrix, *FGF* fibroblast growth factor, *HGF* hepatocyte growth factor, *ICAM-1* intercellular adhesion molecule-1, *IFN-γ* interferon γ, *IL-1Ra* interleukin-1 receptor antagonist, *IL-1 /-6 /-10* interleukin 1 /6 /10, *LIF* leukemia inhibitory factor, *MMPs* matrix metalloproteinases, *MPCs* mesenchymal chondro-progenitor cells, *OA* osteoarthritis, *PAI* plasminogen activator inhibitor, *PDGF* platelet derived growth factor, *PGE2* prostaglandin-E2, *ROS* reactive oxygen species, *SDF-1* stromal cell-derived factor 1, *SEA* N-stearoylethanolamide, *SERPINE* serine proteinase inhibitor, *TGF-β* transforming growth factor β, *TIMP-1 /-2* tissue inhibitor of metalloproteinases-1 /-2, *TNFα* tumour necrosis factor α, *VEGF* vascular endothelial growth factor, *VIM* vimentin

As was mentioned above, several factors have been identified in the composition of CM derived from distinct populations of cells, one being a group of extracellular matrix metalloproteinases, MMP-2, MMP-9, MMP-13 and MMP-19. These proteins belong to the family of zinc-containing proteolytic enzymes and their role is to remodel the ECM of surrounding tissues [66, 123].

MMP-2, also known as gelatinase A, catalyses the breakdown of elements that form the extracellular matrix and promotes cell attachment, proliferation, differentiation processes and programmed cell death [67]. MMP-2 also regulates the activity of one of the most important growth factors, TGF-β, whose disrupted signalling pathways cause the destruction of cartilage tissue and the development of osteoarthritis [68]. Researchers emphasise, however, that the presence of MMP-2 is essential because they drive tissue remodelling with their ability to recruit cells, including osteoblasts, and are involved in inflammatory processes [66, 69]. MMP-9 brings similar properties. Its expression in cartilage is increased in patients with OA [124]. MMP-9 is mainly responsible for recruiting osteoclasts into the hypertrophic zones of the cartilage plate, thus allowing bone formation [68].

In contrast, MMP-13 is a collagenase that cleaves collagen types I, II and III. It digests both cellular matrix and non-cellular components [72]. Its properties, mean that it is involved in cartilage breakdown in OA [83]. Another component, MMP-19, is involved in the degradation of the cartilage cell matrix, aggrecan, Cartilage Oligomeric Matrix (COMP) proteins, types I and IV collagen, laminin, fibronectin and nidogen, a glycoprotein that is part of basic membranes [73, 74].

Among other components of CM are inhibitors of the metalloproteinases mentioned above, TIMP-1 and TIMP-2. These proteins are characterised by anti-fibrotic activity and may prevent cartilage degradation [43, 76]. TIMP proteins have also been attributed to the suppression of pain, which is explained by the reduction of mechanical and thermal hypersensitivity after nerve injury through inhibition of the activity of MMPs [77, 78].

Researchers highlight another component, vimentin (VIM) in the CM may play a role in the chondrogenic differentiation of adult mesenchymal chondro-progenitor cells (MPCs). It is also required adequately to express cartilage genes and regulate the synthesis of ECM’s in chondrocytes and MPCs undergoing chondrogenesis [79, 80]. Bobick et al. demonstrated that inhibition of vimentin expression resulted in a reduction of the expression of the cartilage markers aggrecan, type II collagen, SRY-Box Transcription Factor 5 (SOX5), SRY-Box Transcription Factor 6 (SOX6) and SRY-Box Transcription Factor 9 (SOX9) [79]. A similar relationship was demonstrated by Blain et al. suggesting that the intermediate filament network of vimentin contributes to the maintenance of the chondrocyte phenotype and that its disruption may lead to the development of OA [80].

Serine proteinase inhibitor (SERPINE) family proteins, such as vimentin, maintain the structure of cartilage, but at the tissue level, perform a more precise function in preventing cartilage breakdown. SERPINE1 is involved in the remodelling of tissue and the formation of fibre, due to its ability to inhibit plasmin and cellular matrix metalloproteinases [82]. SERPINE2 inhibits the expression of MMP-13 and reduces cartilage breakdown caused by metalloproteinase [83].

Among the most commonly mentioned growth factors are TGF-β, VEGF, HGF, FGF, and PDGF. Growth factors of the TGF-β family play a key role in every aspect of the tissue development, including chondrogenesis. They control the proliferation, differentiation, and maintenance of the chondrocytes that form cartilage. They also act as inhibitors of the hypertrophic process and reduce oxidative stress [84, 85].

Another component widely described in CM composition is VEGF, a protein responsible for the proliferation and migration of endothelial progenitor cells and angiogenesis. It plays a crucial role in the late stages of chondrogenesis, enabling infiltration and the formation of blood vessels [92–94]. Increased VEGF levels correlate with the progression of OA, specifically with Kellgren and Lawrence (K&L) classification [115]. VEGF signalling has been reported to be involved in OA pain as a growth factor-mediated angiogenesis induction. It increases the inflammatory process and the sensitivity of pain receptors [95].

The other growth factor, HGF, has a significant impact on overall cell survival and proliferation, cellular matrix metabolism, induction and enhancement of the expression of MMPs, and the enhancement of Th2-mediated immune responses [97, 125, 126]. In the context of the osteochondral system, it plays a significant role in osteochondral turnover, bone remodelling and the regeneration of osteoarticular tissue [97, 127].

FGF acts as a mechano-transducer by binding to heparan sulphate proteoglycan chains in the ECM [102]. It is reported that it may also be released in higher concentrations in response to damage to cells [128]. Increased FGF release leads to activation of the extracellular-signal-regulated kinase (ERK) signalling pathway in cartilage [103]. Researchers highlight the chondroprotective properties of FGF due to the factor's ability to inhibit A Disintegrin and Metalloproteinase with Thrombospondin motifs-5 (ADAMTS-5) -mediated aggrecan degradation [102]. It is also thought that FGF induces the synthesis of TIMP-1, MMP-1 and MMP-3, influencing cartilage protection and inhibiting the degradation of its components [103]. FGF also regulates neurogenesis and protects neurons from degeneration after injury, which implies functions in tissue remodelling and repair processes that may be observed against in tissues, such as cartilage [128].

PDGF is a growth factor known for its beneficial properties in healing wounds by affecting chemotaxis, angiogenesis and cell mitosis [104]. It has also been established that PDGF inhibits inflammatory processes and apoptosis in in vitro models by reducing the expression of apoptotic proteins protein 38 (p38), BCL2 Associated X (Bax) and caspase-3 with a parallel increase in the expression of the protein responsible for cell survival, ERK [105]. In chondrogenesis it increases the level of type II collagen that builds cartilage, reduces the level of collagen X in hypertrophic chondrocytes and alleviates cartilage hyperplasia [105].

Other CM components include chemokine, C–C Motif Chemokine Ligand 2 (CCL2) and cytokine, LIF. CCL2 plays the role of chemoattractant for monocytes, macrophages and activated T lymphocytes involved in the inflammatory response [89]. In patients with OA, CCL2 levels are significantly increased in the blood and synovial fluid, suggesting a correlation with the pathogenesis of the disease [129]. The use of this factor as a marker for the diagnosis and treatment of OA is highlighted in some studies [89].

LIF is present in the fluid of patients with arthritis, both rheumatoid arthritis and osteoarthritis [98]. It is involved in the resorption of proteoglycans in cartilage, inflammatory, and catabolic processes. It is thought to be a mediator in the pathogenesis of arthritis [98–100]. The pleiotropic actions of LIF include the ability to regulate cell differentiation, acute phase protein synthesis, stimulation of calcium ion release in bone, proteoglycan resorption and immune response, but are by no means limited to these [101].

Another factor, whose elevated levels may be observed in patients with OA and rheumatoid arthritis (RA), and which is present in CM, is interferon γ (IFN-γ)[81, 130]. It is also known as type II interferon and is produced by T lymphocytes and NK cells. It tends to be responsible for communicating immune cells and activating the body's defence mechanisms. In the context of OA, it contributes to the M1 polarisation of macrophages, which produces reactive oxygen species (ROS) and pro-inflammatory cytokines. These processes are associated with the progression of osteoarthritis [106]. T cells producing the above cytokine also induce osteoclastogenesis by expressing Receptor Activator for Nuclear Factor κβ Ligand (RANKL), leading to bone resorption [107].

Like LIF, the dualistic nature, pro-inflammatory and anti-inflammatory, is demonstrated by IL-6. The pro-inflammatory functions of interleukin are associated with the induction of intercellular adhesion molecule-1 (ICAM-1) expression, downregulation of type II collagen expression and the maintenance of acute inflammation or conversion to a chronic form [98, 111]. IL-6 is also associated with the immune response, as it induces differentiation of B lymphocytes, making these cells capable of producing antibodies [112]. It is also an important factor in homeostasis in bone insofar as it increases the expression of RANKL, which induces osteoclast differentiation and leads to excessive bone resorption [113]. IL-6 influences RA development by participating in vascular inflammation pathogenesis. It is associated with excessive angiogenesis and vascular permeability caused by increased VEGF expression [114]. Researchers suspect that IL-6 may modulate the balance between MMPs and TIMPs. Disruption of this balance leads to the degradation of articular cartilage and the development of osteoarthritis [98].

Interleukin 10 (IL-10), like IL-6, is elevated in patients with OA. It tends to perform anti-inflammatory functions by inhibiting proinflammatory factors such as IL-1β, TNF and MMPs. It also upregulates interleukin-1 receptor antagonist (IL-1Ra) and TIMP [111, 115]. IL-10 exhibits chondroprotective activity by reducing the apoptosis of cartilage tissue cells and counteracting pro-inflammatory IL-1 [115, 116].

Stromal cell-derived factor 1 (SDF-1) or C-X-C motif chemokine 12 (CXCL12) belongs to the group of pro-inflammatory chemokines. It is present in the synovial membranes and synovial fluid of OA patients and also in subchondral bone [117, 131]. It tends to be produced by BM-MSCs and osteoblasts, whereas chondrocytes in healthy tissue do not express this factor [131]. SDF-1 binds to its ligand, C-X-C chemokine receptor type 4 (CXCR4), which conjugates to a G-protein on the surface of chondrocytes, allowing the activation of multiple signalling pathways [117, 131] responsible for apoptosis and release of MMP, increased expression of IL-1, TNFα [118, 119]. It has been credited with a role in the degradation of cartilage and the enhancing of the inflammatory process in OA by reducing the expression of type II collagen and aggrecan [117–120]. SDF-1 intraarticular injection has been shown to induce the OA model in the knee in rabbits more efficiently than traditional surgical methods [119]. It has been stressed that treatment targeting the SDF1/CXCR4 signalling pathway may be a promising therapeutic method for the treatment of arthritis [118].

The ingredient responsible for the ageing of chondrocytes by the upregulation of MMPs and inflammatory factors such as SDF-1 and VEGF is Dickkopf 1 (DKK-1) [108, 109]. It is a growth factor and antagonist of the canonical Wnt/β-catenin signalling pathway. DKK-1 is thought largely to regulate bone remodelling in arthritis [108, 110]. The Wnt/β-catenin signalling pathway plays a role in bone resorption, and joint degradation in the pathogenesis of RA [108, 109]. The expression of DKK-1 is increased in RA patients in synovial fluids and synovial fibroblasts [108].

**Fig. 2:**
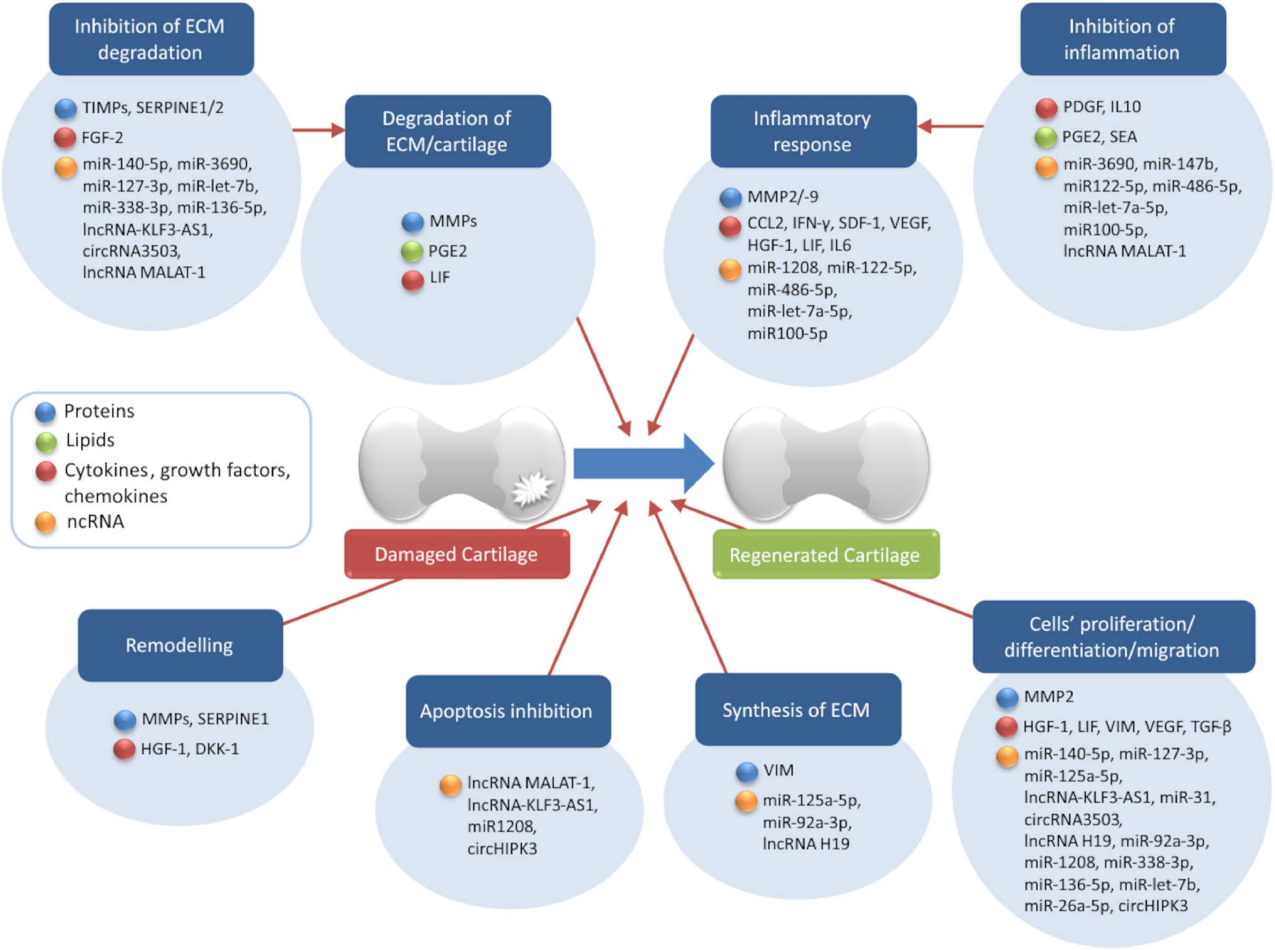
CM components and their effect on articular cartilage biology. Distinct factors included in conditioned media as free molecules or delivered by extracellular vesicles can affect several biological processes, such as the interplay between regeneration/degradation of ECM, proliferation, migration of chondrocytes, and modulation of local inflammation response

Among the components of the CM were detected a number of lipids, which are involved in the development of inflammatory processes, prostaglandin-E2 (PGE2) and N-stearoylethanolamide (SEA). PGE2 may play both pro-inflammatory and anti-inflammatory roles, depending on its concentration [64]. It induces macrophages to produce the anti-inflammatory IL-10, reduces apoptosis in hepatocytes and reduces the expression of plasminogen activator inhibitor (SERPINE1/PAI)-1 mRNA [86]. PGE2 is present in articular cartilage with inflammatory arthropathy [82], downregulates aggrecan expression and regulates plasmin activator secretion and proteinase expression, which may lead to extracellular matrix degradation [82]. SEA, in contrast, is a lipid of endogenous origin, of the N-acylethanolamides family, which has anti-inflammatory and immunomodulatory properties [64]. It is thought to be involved in downregulating the expression of many pro-inflammatory factors, but its exact role in OA is not fully understood [64, 122]. The effect of the listed factors on articular cartilage is summarised in Fig. 2.

## Effect of Conditioned Media from Mesenchymal Stromal Cells on OA Chondrocytes

MSCs are the most popular source of cells for use in regenerative medicine, due to their availability and wide clinical application in different fields of medicine. Several clinical trials regarding their use are taking place [132]. As mentioned above, their unique paracrine parameters provide the beneficial effect of stromal cells in tissue regeneration processes [64, 65]. Stromal cells produce exosomes and growth factors that may hinder degradation processes and induce tissue regeneration [133]. The MSC secretome, CM containing factors, exosomes and microvesicles, also reduces inflammation and boosts regeneration processes, rendering it a promising option for OA therapy [134]. A significant advantage of using CM over cells to treat OA is that treatment with CM requires no surgery, but a simple ambulatory procedure [87]. This advantage entails quicker recovery time for the patient and also entails with financial benefits. For CM to be applied to OA therapy, however, standardisation of protocols for obtaining and harvesting the culture medium is needed, as well as a precise definition of its composition [29]. Table 3 summarises recent advances in the field of the application of CM in OA management with its various detailed composition of CM and potential active agents.

**Table 3:** Sample protocols including conditioned medium in the treatment of OA chondrocytes

MSC source	CM producing	Subject	Experimental conditions	Outcome: CM impact	Ref
human ASC	- base: complete serum-free DMEM; - cells seeded 1 × 10^5^ per well; - collection time: after 2 and 7 days (pooled)	- chondrocytes and synoviocytes from patients with OA K&L = 3 or 4	- time: 7 days; - DMEM:CM ratio: 75:25; 50:50; 25:75	- no decrease in IL6, MCP-1, MIP1-α, RANTES expression (in contrast with coculture); - no decrease in IL8, GROα expression in synoviocytes; - partially decreased IL8, expression of GROα in chondrocytes; - less efficient reduction in expression of MMP-13, ADAMTS4, ADAMTS5 and increase in the expression of TIMP-1, TIMP-3 (in contrast with coculture); - CM contains a low concentration of PGE2 to reduce inflammation	[135]
human AD-MSC	- base: DMEM/F12; - cells seeded 2 × 10^6^; - cells at 80–90% confluence	- chondrocytes and synoviocytes from patients with OA K&L = 4	- DMEM/F12 with 1 ml CM; - coculture of chondrocytes and synoviocytes induced with IL-1β	- reduction of GAGs concentration; - reduction in NO production; - reduction in expression of IL1β, MMP-13, ADAMTS5 in cartilage; - reduction in expression of MMP-13 and ADAMTS5 in synovium; - enhancement in expression of TIMP-1 in cartilage and synovium	[136]
human ASC	- base: complete serum-free DMEM; - cells after the 4^th^ passage; - cells at between 80 and 90% confluence	- chondrocytes from patients with OA	- time: 24 h, 48 h, 72 h; - complete DMEM with CM at a ratio of 1:5; - with/without TNFα stimulation	- blunting of hypertrophy; - reduction of osteocalcin, COL10, MMP-3, MMP-13 activity; - CM contains chondroprotective factors such as OPG and DKK-1	[137]
human ASC	- base: DMEM/F12 with 15% human serum (AB-blood group donors); - cells before and after first passage; - cells at semi-confluence	- chondrocytes from patients with OA	- time: 1–7 days; - DMEM/F12 with 10% human serum with/without 1 ml of CM for 6-well plates or 2 ml of CM for 3.5 cm plates; - cells stimulated with IL-1β	- reduction of SA-β-gal; - reduction of γH2A.X foci; - reduction in formation of actin stress fibres; - reduction in production of oxidative stress and protein kinases; - reduction in expression of caveolin-1 and p21; - reduction of p53 acetylation	[138]
human WJ-MSC	- base: serum-free DMEM; - cells after the 4^th^ passage; - cells at 70% confluence; - collection time: after 48 h	- chondrocytes from OA patients	- time: 3 and 6 days; - DMEM/FBS with WJ-MSC-CM	- upregulation in expression of COL2A1, ACAN, COMP, and SOX9 after 3 and 6 days in monolayer and mass culture; - reduction in expression of chondrocyte-specific genes after 6 days in monolayer; - maintenance of high expression of chondrocyte-specific genes after 6 days in mass culture	[49]
human WJ-MSC	- base: complete medium; - cells seeded 3 × 10^5^ per well; - cells treated with IGF1 (0 or 150 ng/ml) for 7 days	- chondrocytes CHON002 (ATCC CRL-2847)	- time: 7–14 days; - 15% or 30% CM in medium; - after CM, cells treated with IL-1β for 5 days	- increase in expression of COL2 in 15% IGF1-WJMSC-CM; - reduction in concentration of inflammatory cytokines; - reduction in expression of MMP-3	[47]
human SM-MSC	- base: complete medium; - cells seeded 5 × 10^5^ per flask; - cells at 80% confluence; - cells treated with IGF1 (0 or 150 ng/ml) for 7 days	- chondrocytes CHON002 (ATCC CRL-2847)	- time: 7 days; - 15% or 30% CM in medium; - after CM, cells treated with IL-1β for 5 days	- increase in expression of SOX9, and COL2; - reduction in expression of COL10, MMP-13, ADAMTS4; - IGF1-preconditioning improves the effect of CM in lowering hypertrophic factors and raising anti-hypertrophic factors	[139]
human SHED	- base: serum-free DMEM/F12; - cells seeded 5 × 10^3^ per cm^2^ until 70% confluence; - collection time: after 48 h or 72 h	- chondrocytes from patients with OA	- time: 48 h; - cells treated with CM collected after 48 h or 72 h; - cells stimulated with IL-1β	- increase in the concentration of anti-inflammatory factors; - increase in expression of COL2 and ACAN; - decrease in expression of MMP-13 and NF-κB	[31]
human BM-MSC	- base: serum-free DMEM w/o antibiotics; - cells after the 3^rd^ passage; - cells at 70% confluence; - collection time: after 48 h	- chondrocytes from rat model of OA (in vivo)	- time: 8 weeks; - weekly CM injection (100 µl)	- well-preserved subchondral bone structure; - more abundant cartilage matrix; - reduced MMP-13/TIMP-1 ratio; - inhibited chondrocyte apoptosis with increased autophagy	[140]

*ACAN* aggrecan, *ADAMTS-* A Disintegrin and Metalloproteinase with Thrombospondin motifs, *AD-MSCs* adipose tissue-derived MSCs, *ASCs* adipose stromal cells, *BM-MSCs* bone marrow MSCs, *CM* conditioned media, *COL10* collagen type X, *COL2A1* collagen type II α 1 chain, *COMP* cartilage oligomeric matrix protein, *DKK-1* dickkopf 1, *DMEM* Dulbecco's Modified Eagle Medium, *DMEM/F12* Dulbecco's Modified Eagle Medium/Nutrient Mixture F-12, *GAGs* glycosaminoglycans, *GROα* growth-regulated α protein, *IGF-1* insulin-like growth factor 1, *IL-1β /-6 /-8* interleukin 1β /6 /8, *K&L* Kellgren and Lawrence, *MCP-1* monocyte chemotactic protein 1, *MIP1-α* macrophage inflammatory protein 1α, *MMPs* matrix metalloproteinases, *MSC* mesenchymal stromal cell, *NF-κB* nuclear factor kappa-light-chain-enhancer of activated B cells, *NO* nitric oxide, *OA* osteoarthritis, *OPG* osteoprotegerin, *p21 /p53* protein 21 /53, *PGE2* prostaglandin-E2, *RANTES* regulated upon activation, normal T cell expressed and presumably secreted, *SA-β-gal* senescence-associated β-galactosidase, *SHED* human exfoliated deciduous teeth stromal cells, *SM-MSCs* synovial membrane MSCs, *SOX9* SRY-Box transcription factor 9, *TIMP-1 /-2* tissue inhibitor of metalloproteinases-1 /-2, *TNFα* tumour necrosis factor α, *WJ-MSCs* Wharton's jelly MSCs

In their study Manferdini et al., compared the effects of CM and the coculture system on chondrocytes. Adipose stromal cells (ASCs) taken from healthy patients who undergo liposuction of subcutaneous abdominal fat were used to obtain CM and coculture. CM was harvested from cells (seeded at 1 × 10^5^ per well) on the 2^nd^ and 7^th^ day after administration of complete serum-free Dulbecco's Modified Eagle Medium (DMEM) to cells in culture and then pooled. Chondrocytes and synoviocytes were collected from OA patients showing K&L 3 or 4. Cells were then cocultured with ASC and treated with a CM for 7 days. The study failed to show that culture with CM led to a reduction in expression levels of IL-6, monocyte chemotactic protein 1 (MCP-1), macrophage inflammatory protein-1 α (MIP1-α) and regulated upon activation, normal T cell expressed and presumably secreted (RANTES) in the cells examined compared to the coculture system. The culture with CM also failed to reduce interleukin 8 (IL-8) and growth-regulated α protein (GROα) levels in synoviocytes, but partially reduced chondrocytes. The use of CM showed a less effective reduction in MMP-13, ADAMTS4 and ADAMTS5; an increase in TIMP-1 and TIMP-3, rather than the coculture of cells with ASC. The CM contains too low a concentration of PGE2 to reduce inflammatory processes in chondrocytes and synoviocytes. It was therefore concluded that ASC-CM displays a limited ability to reduce inflammation and down-regulate proteases that contribute to ECM degradation [135].

Mendia et al. compared the effects of CM and HA on the coculture of chondrocytes with synoviocytes. CM was obtained from human AD-MSCs taken from total or partial hip arthroplasty patients and stimulated with IL-1β for 24 h. Stimulation with IL-1β triggered ECM degradation and activation of mediators and effectors of progressive cartilage loss. AD-MSCs were seeded 2 × 10^6^ in Dulbecco's Modified Eagle Medium/Nutrient Mixture F-12 (DMEM/F12), and the medium was harvested when cells reached between 80 and 90% confluence. The results were compared for the coculture of IL-1β-induced cells cultured in DMEM/F12 with HA or CM. CM was found to lead to a reduction in glycosaminoglycans (GAGs) levels, and HA. A reduction in nitric oxide (NO) production was also only confirmed in the sample with the addition of CM. Culture with CM also reduces the expression of IL-1β, MMP-13 and ADAMTS5 in cartilage and the expression of MMP-13 and ADAMTS5 in the synovium. In contrast, HA only reduces MMP-13 and ADAMTS5 levels in cartilage and ADAMTS5 in the synovium. CM, like HA, leads to the upregulation of the expression of TIMP-1 in both cartilage and synovium. This demonstrates that both HA and AD-MSC-CM show beneficial effects in OA therapy, but the impact of CM is more prominent [136].

As in the studies described above, Niada et al. used stromal cells derived from adipose tissue. ASCs were collected from healthy patients undergoing aesthetic or prosthetic surgery. The medium was obtained from cultured cells after the 4^th^ passage showing between 80 and 90% confluence after being cultured in serum-free DMEM for 72 h in starving conditions. Cartilage tissue cells were collected from patients undergoing total hip replacement from femoral heads. Chondrocytes in culture were initially stimulated with TNFα to induce hypertrophy; then with or without ASC-CM in a 1:5 ratio with DMEM supplemented with 1% fetal bovine serum (FBS) for 24 h, 48 h or 72 h. The results highlighted that CM blunted TNFα-induced hypertrophy and reduced osteocalcin, collagen type X (COL10), MMP-3 and MMP-13 activity. ASC-CM also contained chondroprotective factors such as osteoprotegerin (OPG), and DKK-1. The study established that CM exhibits anti-hypertrophic and anti-catabolic effects [137].

Platas et al., a group of researchers who also used stromal cells derived from adipose tissue, used cells obtained during abdominoplasty. The cells were cultured before or after the 1^st^ passage to achieve semi-confluence. During culture, the medium used was DMEM/F12 supplemented with antibiotics and 15% human serum, derived from donors with AB blood group. 48 h after the beginning of the culture, the medium was collected from above the cells and pooled. Chondrocytes were obtained from the knee cartilage of patients with advanced osteoarthritis. OA-specific inflammation was induced in the cells with IL-1β and then cultured for between 1 and 7 days in complete DMEM/F12 with or without CM. 1 ml ASC-CM for 6-well plates or 2 ml for 3.5 cm plates was added. CM reduced markers of IL-1β-induced senescence-like accumulation of yH2A.X foci and the formation of actin stress fibres. CM also reduced the production of oxidative stress, reduced the activation of myogen-activated protein kinases, downregulated the expression of caveolin-1 and protein 21 (p21), and also reduced protein 53 (p53) acetylation. These results concluded that ASC-CM shows a protective effect in degenerative joint conditions and could be used as a potential therapy for OA [138].

Adipose tissue cells and those from the umbilical cord have been used to obtain CM for OA therapy. In their study Famian et al. isolated WJ-MSCs from the umbilical cord of women who had undergone a caesarean section. MSCs were cultured in serum-free DMEM until the 4^th^ passage, and then the medium was harvested when the cells reached 70% confluence. The effect of CM was tested on chondrocytes collected from patients undergoing joint replacement for femoral neck fractures. Chondrocytes were cultured until the 3^rd^ passage and then seeded at 1 × 10^6^ in both monolayer and mass culture. The cells were then cultured in DMEM medium with FBS alone or with the addition of WJ-MSC-CM for between 3 and 6 days, and analyses were performed. It was demonstrated that the expression of the cartilage genes collagen type II α 1 chain (COL2A1), aggrecan (ACAN), COMP and SOX9 was significantly upregulated after the 3^rd^ and the 6^th^ day of culture in both monolayer and mass cultured cells. After 6 days of culture with CM, however, a decrease in the expression of the above-mentioned genes was observed in monolayer cultured cells, while cells in mass culture continued to show high gene expression. The results presented by Famian et al. indicate that WJ-MSC-CM has a beneficial effect on cartilage gene expression in chondrocytes cultured in both monolayer and mass cultures. The long-lasting effect is, however, only seen in cells cultured in the mass culture system [49].

The research group of Kusuma et al. also used WJ-MSC to obtain a CM. The stromal cells, isolated for previous studies [141], were seeded at 3 × 10^5^ cells per well in a complete medium. Some MSCs were induced with insulin-like growth factor 1 (IGF-1) (150 ng/ml) for 7 days and CM was then harvested. The stromal cells were induced to secrete more factors to stabilise the chondrocyte phenotype and lead to more efficient ECM production. The cartilage tissue cells also came from a commercial source and were CHON002 chondrocytes (ATCC CRL-2847). Chondrocytes were seeded with 5 × 10^5^ cells per T-25 flask and cultured in the presence of CM (15% or 30%) for 48 h. After a time, the CM was removed and cells were treated with IL-1β for 5 days to induce an OA-specific phenotype. The researchers highlighted that the most significant increase in the expression of type II collagen was observed in the trial in which WJ-MSCs were initially induced with IGF-1 and CM was applied at a concentration of 15%. The study demonstrated a beneficial effect of WJ-MSC-CM on the reduction of anti-inflammatory cytokines, such as TNFα and IL-10, as well as the reduction in the expression of MMP-3 in OA chondrocytes [47].

The same research group published a similar study a year later, in which CM was derived from human SM-MSCs. Stromal cells were collected from patients with OA with K&L 4 during knee joint biopsies. They were then seeded at 5 × 10^5^ per flask in a complete medium, and CMs were harvested when the cells reached 80% confluence. MSCs were also induced with IGF-1 for 7 days. In the initial stages of the study, cell induction was shown to be most effective using IGF-1 at a concentration of 150 ng/ml, which was used in the following stages of the study. As in the study by Kusuma et al., the effect of CM was tested on cell line CHON002 [47]. Chondrocytes were seeded 1 × 10^6^ cells per flask in complete DMEM supplemented with 15% or 30% SM-MSC-CM and, after removal of the medium, cells were induced with IL-1β. It has been shown that preconditioning SM-MSCs with IGF-1 leads to a more significant decrease in hypertrophy markers and an increase in those factors that are thought to prevent chondrocyte hypertrophy. The IGF-1-SM-MSC-CM increases the expression of SOX9 and COL2 with a parallel reduction in the expression of COL10, MMP-13 and ADAMTS4, hypertrophic markers. Previous studies suggest a more beneficial effect of medium-conditioned neural stromal cells in chondrocyte cultures than those exhibiting an OA-specific phenotype [139].

Muhammad et al. detail another source from which MSCs were obtained for CM. The researchers isolated human SHED cells from deciduous molars and seeded them at 5 × 10^3^ per cm^2^ and cultured them in serum-free DMEM until 70% confluence was achieved. When the appropriate confluence was reached, the medium in the cells was changed and CM was harvested after 48 h or 72 h. The effect of the CM was studied on chondrocytes collected from OA patients undergoing total knee arthroplasty. Chondrocytes (1 × 10^4^ cells/cm^2^) were initially stimulated with IL-1β and then treated with SHED-CM. The CM was shown to increase the concentration of anti-inflammatory factors and stimulate the expression of hyaline cartilage markers, COL2 and ACAN, as well as leading to a reduction in the expression of MMP-13 and nuclear factor κB (NF-κB), a mediator of pro-inflammatory and catabolic factors. This suggests a beneficial effect of CM on OA chondrocytes and potential in regenerating ECM proteins and mitigating inflammation induced by the upregulation of the expression of NF-κB and MMP-13 [31].

BM-MSCs are also a potential source of CM. They were used by Chen et al. in their study. The research was designed to evaluate the effects of BM-MSC-CM on the rat model of OA induced by the anterior cruciate ligament transaction and destabilisation of the meniscus. Human BM-MSCs obtained from ScienCell Research Laboratories were cultured until the 3^rd^ passage. Once cells reached 70% confluence, the culture medium was changed to serum-free medium w/o antibiotics and pooled after 48 h. CM was administered to rats in the form of intraarticular injection. The analyses carried out confirmed the overall alleviating effect of symptoms and inconvenience associated with the development of OA after the application of CM. A morphologically well-preserved subchondral bone structure was confirmed in those animals receiving CM, along with more abundant cartilage matrix. The use of a CM also inhibited rats’ chondrocytes apoptosis. The immunohistochemical staining showed a reduced ratio of MMP-13 to TIMP-1, confirming the beneficial effects of MSC-CM in OA management [140].

Despite the different sources of MSCs, most studies and protocols presented support the thesis that the beneficial effects on cartilage tissue cells in in vitro culture may be achieved with stromal cell-conditioned media. These beneficial effects may include the inhibition of hypertrophy, oxidative stress, ageing, the degradation of ECM, the upregulation of chondrogenic-related genes and the secretion of chondroprotective factors. Due to its properties, MSC-CM shows potential for application in OA therapy. To do this, however, it is necessary to develop appropriate protocols for obtaining CM based on a serum-free system, so that an excessive immune response in the patient is avoided.

## Exosomes are an Essential Component of the Conditioned Medium

Besides freely excreted molecules, highly organised membranous structures, commonly called extracellular vesicles (EVs), could be found among the stem cell secretome [142, 143]. We can distinguish among them (depending on the origin and size) microvesicles/ectosomes (surface of the cell; 100 – 1000 nm), apoptotic bodies (cells undergoing apoptosis; 50 – 5000 nm) and exosomes (internal compartments of the cells; 30–150 nm) [144]. A vast majority of the literature is focused on the properties and functionality of the exosomes – the endosomal-origin bilipid layer structures with cup-like shape morphology [145]. They already have gained an undoubtful place in cancer biology, acting as a potential source of biomarkers in early detection and prognostic purposes [146–148]. Cancer cells use them to manipulate the immune system and local/distal tissue environment to form a metastatic niche [149, 150]. They have strong immunomodulatory properties, are responsible for immune tolerance and are crucial players in intracellular communication in local and distal areas of the organism [151]. They have a unique composition of lipids, proteins and nucleic acids (the main source of miRNA, lncRNA), which could be modified in response to physical or chemical stimulation, acting as an indicator of environment changes [152, 153].

Exosomes have gained considerable interest in their applications as a drug delivery platform since their structure enable the protection of molecular cargo [143, 154]. Additionally, modification of their composition through overexpression of particular molecules (especially miRNA packaging) could lead to the formation of tools for targeted therapy [143, 155]. They are unable to proliferate, exhibit properties of parental cells, and are highly tolerable by the recipient, could cross the blood–brain barrier, which makes them almost a universal solution for regenerative medicine purposes [156–158]. Since exosomes are dependent on the physiological state of the parental cell, the standardisation of their isolation protocol is still needed to be established, as well as cell source, culture system, the medium supplementation with appropriate growth factors/chemical compounds, and knowledge about their exact molecular profile [159–162]. One of the critical roles in their harvesting is formulation of the medium, which should be serum- and xeno-free, due to the vast amounts of EVs from animal origin. Consequently, their cargo profile could also change [163]. Besides these facts, their unique protective and immunomodulatory properties has found potent usefulness in a few fields of regenerative medicine, such as attenuation of the effect of myocardial infarction [164], kidney ischemia [165], brain ischemic stroke [166], improved regeneration with limited scarring during wound healing [167], increases angiogenesis [168], reduction of lung fibrosis caused by the biological or chemical factor [60, 169], diminishing the damages of nucleus pulposus or temporomandibular joints [170–173]. Several studies have also implied that exosomal cargo is a crucial player in the modulation of the chondrogenesis of MSC, depending on the source of EVs [174, 175].

In this part of the review, we would like to focus on their potent application in managing damaged large joints affected by OA. In recent years, many studies regarding using exosomes isolated from distinct MSC sources in the management of musculoskeletal diseases have been published [176]. They were likely ideal candidates as a delivery platform for the several non-coding RNA with therapeutic potentiation [177]. It was shown that they were able to modify the local environment through the attenuation of inflammation by internalising their cargo into macrophages (causing their polarisation into M2 type) and synoviocytes (limiting their inflammatory-related secretome) [178–180]. Most importantly, EVs derived from MSC could diminish and reduce the impact of the OA induced chemically (IL-β, TNF-α or collagenase type II) or via mechanical damages on chondrocytes biology [179, 181–184]. It was assessed by maintaining significantly high expression of markers related to hyaline cartilage (i.e. COL2A1, SOX9, ACAN) with simultaneous downregulation of markers responsible for chondrocytes maturation/hypertrophy and ECM degradation (i.e. MMP13, ADAMTS, RUNX2) [45, 185, 186]. Exosomes also positively impacted the migration, proliferation, and survival of chondrocytes, which caused the slower degradation of ECM, reduced tissue depletion and slower disease progression of OA-affected joints in *in* vivo models [185, 187–189]. These processes were partially regulated via the NF-κB signalling pathway, a crucial one in the inflammatory response in OA, and the WNT/β-catenin signalling pathway, which is responsible for chondrogenic differentiation and chondrocytes catabolic/anabolic homeostasis [154, 162, 177, 186, 190]. Interestingly, among these studies, there was no mention of the negative impact or side effects, which could decline their usage in future clinical trials. However, there was no common mechanism which could provide a universal solution. Studies presented below gave information about the partial protective properties of EVs derived from MSC, but none of them was able to provide full-thickness recovery. As suggested, their usage should be limited to the early stages of OA. The outcomes of the latest studies regarding their use in the management of OA are summarised in Table 4 and molecules involved in OA biology are presented in Fig. 2.

**Table 4:** The effect of exosomes isolated from the distinct MSC sources on the prevention/modification of OA

Source of cells	In vitro	In vivo	Ref
SM-MSC (Human)	Exosomes derived from overexpressing miR-140-5p SM-MSC and WT SM-MSC have increased migration and proliferation. The presence of miR-140-5p reduced the degradation of ECM caused by WNT5a/b carried in SMSC exosomes	Rat; 12 weeks; No adverse effect was observed. The progression of OA was significantly delayed in the group treated with exosomes derived from SMSC overexpressing miR-140-5p *vs* exosomes derived from non-modified cells	[177]
BM-MSC (Mouse)	Exosomal miR-3960 has led to decreased degradation of ECM and inflammation of OA chondrocytes through regulation of the PHLDA2/SDC1/Wnt/β-catenin axis	Mice; 7 weeks; No adverse effect was observed. The delayed progression of OA and decrease of proinflammatory markers were highly reduced after exposure to MSC-EVs-miR-3960	[191]
BM-MSC (Human)	hBMMSC-EXOs have increased the viability, migration, proliferation and mitochondrial function in chondrocytes	Rabbit; 5 weeks; No adverse effect was observed. The osteochondral defect was partially repaired	[192]
BM-MSC (Human)	The exosomes derived from BMSC have an impact on macrophages polarisation from M1 to M2 and neutralise their effect on chondrocytes hypertrophy after co-culture	Rat; 4 weeks; No adverse effect was observed. The BMSC exosomes decreased hypertrophic markers and reduced the formation of osteophytes in the damaged joint area. Additionally, the infiltration of synovial macrophages was weakened	[178]
UC-MSC (Human)	UC-MSC exosomes increase the proliferation and migration of chondrocytes. They protect chondrocytes from IL-1β induced apoptosis and maintain their phenotype	Rat; 4 weeks; No adverse effect was observed. The injected exosomes isolated from UC-MSC prevented damaged cartilage from developing OA and promoted the expression of anti-inflammatory factors. They caused the enrichment of the M2 macrophages population	[193]
MSC (Human)	Exosomes derived from MSC overexpressing lncRNA malat-1, inhibited the apoptosis and inflammatory markers related to OA in treated chondrocytes better than exosomes from non-modified MSC. They also increased the proliferation and migration of chondrocytes	Rat; 6 weeks; No adverse effect was observed. The malat-1 exosomes had better protective properties and less degraded ECM in OA-induced damages of the rat knee compared to exosomes from non-modified MSC	[194]
BM-MSC (Rat)	Exosomes enriched with miRNA-127-3p were responsible for diminished damage and apoptosis of chondrocytes exposed to IL-1β by targeting the Wnt/β-catenin pathway through downregulation of CDH11	N.D	[195]
BM-MSC (Human)	Exosomal miR-125a-5p significantly impacts chondrocytes migration, proliferation and ECM production through regulation of E2F2 in the traumatic OA	N.D	[184]
MSC (Human)	Exosomes containing lncRNA-KLF3-AS1 have been responsible for regulating GIT1 expression by binding to miR-206. Consequently, its presence led to increased proliferation, maintaining of chondrogenic phenotype and inhibited apoptosis induced by IL-1β	N.D	[182]
SM-MSC (Human)	Isolated exosomes from overexpressing miR-31 SM-MSCs promoted the proliferation and migration of chondrocytes through reduced expression of KDMA2	Mice; 8 weeks; No adverse effect was observed. The exosomes enriched with miR-31 and non-modified one has shown similar protection against OA with mildly reduced inflammation markers	[196]
SM-MSC (Human)	The SMSC containing WNT5a/b and circRNA3503 has promoted migration and proliferation of chondrocytes without loss of ECM proteins. They have shown alone and slowly released in PDLLA-PEG-PDDLA hydrogel the chondroprotective properties against IL-1β induced OA	Rat; 12 weeks; No adverse effect was noticed. The combination of hydrogel with modified exosomes has shown the best protective effect against OA	[188]
UC-MSC (Human)	Mechanical stimulation of UC-MSC enriched exosomal cargo with lncRNA H19, positively affecting chondrocytes viability, proliferation, migration and increased production of ECM	Rat; 4 and 8 weeks; No side effects were notified. The exosomes derived from mechanically stimulated cells had improved the regeneration of damaged cartilage better than the control groups and exosomes with reduced expression of lncRNA H19	[197]
BM-MSC (Human)	miR-92a-3p transported in exosomes promoted proliferation, enhanced expression of matrix-related genes downregulating WNT5a. That miR also enhanced the chondrogenic differentiation of MSC	Mice; 4 weeks; Lack of adverse effect was observed The damage of cartilage was partially inhibited with maintained ECM in the group of exosomes isolated from MSC overexpressing miR-92a-3p	[198]
UC-MSC (Human)	miR-1208 transported in UC-MSC exosomes is responsible for chondrocytes enhanced proliferation, migration and inhibition of apoptosis through downregulation of METTL3. The degradation of ECM was inhibited by the modification of macrophages' inflammation response by modification of NLRP3 activity	Mice; 6 weeks; No adverse effect was observed. The injection of exosomes containing miR-1208 prevented the degradation of ECM and delayed the progression of OA	[187]
BM-MSC (Human)	Exosomes had a stimulatory effect on viability, proliferation, migration and maintenance of the stable chondrogenic phenotype after IL-1β OA induction. Their caused decreased phosphorylation of proinflammatory pathways Erk1/2, PI3K/Akt p38, TAK1 and NF-κB	N.D	[199]
BM-MSC (Human)	After exposure to IL-1β, BM-MSC has secreted exosomes significantly enriched with miR-147b compared to non-treated cells. Consequently, the inflammation of synovial cells was more efficiently diminished by modified EXO. The mechanism involved inhibition of NF-κB pathway	N.D	[181]
UC-MSC (Human)	Exosomes are responsible for M2 macrophage polarisation, enabling them to protect chondrocytes from their apoptosis and decreasing the severity of IL-β induced OA. Their immunomodulatory effect was better after stimuli with exosomes *vs* PRP. The enriched population of several miRNA in UC-MSC exosomes was notified (miR-122-5p, miR-486-5p, miR-let-7a-5p and miR-100-5p)	Rat; 4 and 8 weeks; No adverse effect was observed. Exosomes from UC-MSC have improved knee cartilage restoration and protection better than PRP injections. Polarised and infiltrated M2 macrophages caused the diminished progression of OA	[200]
AD-MSC (Mouse)	Exosomes isolated from AD-MSC overexpressing miR-338-3p, which targets the RUNX2 gene, has promoted the increased proliferation, viability and reduced degradation of ECM of chondrocytes in IL-1β induced OA in comparison with non-modified exosomes	N.D	[180]
BM-MSC (Human)	Exosomes enriched with miR-136-5p have limited the degradation of ECM specific for hyaline chondrocytes and promoted their migration by targeting ELF3, a transcript factor elevated in traumatic OA	Mice; 1 h; No adverse effect was observed. The injection of modified exosomes protected cartilage from severe damage caused by trauma-induced OA	[201]
AD-MSC (Human)	Exosomes positively impacted chondrocytes and synoviocytes exposed to IL-1β by diminishing proteins regulating the NF-κB signalling pathway. As a result, the expression of the proinflammatory and catabolic enzymes was downregulated	N.D	[202]
SMSC (Human)	EVs isolated from SMSC pretreated with LPS has shown better protective properties in chondrocytes exposed to IL-β. They increased migration, motility, proliferation and viability. The exosomal let-7b was responsible for that outcome by targeting ADAMTS5	Mice; 6 weeks; No adverse effect was observed. The EVs from LPS-treated SMSCs protected cartilage from ECM degradation better than EVs isolated from non-modified cells	[203]
UD-MSC (Human)	EVs isolated from hypoxic UD-MSC increased the migration and proliferation of chondrocytes. Their effect was provided by upregulated miR-26a-5p, which modified the expression of PTEN	N.D	[204]
BM-MSC (Human)	Exosomes protected the chondrocytes from TNF-α induced inflammation by inhibition of NF-κB signalling pathway. They enhanced chondrocyte proliferation and production of ECM	N.D	[205]
BM-MSC (Human)	Isolated exosomes have shown the protective effect against IL-1β induced OA by inhibiting apoptosis through regulation of lncRNA LYRM4-AS1/GRPR/miR-6515-5p axis	N.D	[206]
ESC-MSC (Human)	Exosomes isolated from differentiated hESC into MSC has shown their involvement in the protection of chondrocytes exposed to IL-1β by promoting ECM production and reducing catabolic activity	Mice; 8 weeks. No adverse effect was observed. The injected exosomes have shown to decelerate the progression of OA and promote ECM production	[207]
MSC (Human)	EVs containing circRNA HIPK3 are responsible for increased proliferation, migration, expression of chondrogenic markers and viability of chondrocytes. They protect them from OA progression after IL-1β exposure. The mechanism of EVs is related to the regulatory network between circRNA HIPK3/miR-124-3p/MYH9	Mice; N.D.; The injected EVs isolated from circHIPK3 overexpressing and non-modified MSCs have shown protective properties against the OA development with maintenance expression markers characteristic of hyaline cartilage	[185]

*AD-MSCs* adipose tissue-derived MSCs, *BM-MSCs* bone marrow MSCs, *CDH11* Cadherin 11, *circRNA* circular RNA, *E2F2* E2F transcription factor 2, *ECM* extracellular matrix, *ELF3* E74 Like ETS Transcription Factor 3, *ERK1/2* extracellular signal‑regulated protein kinase, *GIT1*, ARF GTPase-Activating Protein, *GRPR* Gastrin Releasing Peptide Receptor, *HIPK3* homeodomain interacting protein kinase 3, *IL-1β* interleukin 1β, *KDMA2* Lysine Demethylase 2A, *lncKLF3-AS1* KLF Transcription Factor 3 Antisense RNA 1, *lncRNA* long-non coding RNA, *LYRM4* LYR Motif-Containing Protein 4, *METTL3* Methyltransferase 3, *miRNA* micro RNA, *MSC* mesenchymal stromal cell, *MYH9* myosin heavy chain 9, *N.D.* No data, *NF-κB* nuclear factor kappa-light-chain-enhancer of activated B cells, *NLRP3* NLR family pyrin domain containing 3, *OA* osteoarthritis, *PDDLA-PEG-PDDLA* Poly(D,L-lactide)-block-poly(ethylene glycol)-block-poly(D,L-lactide), *PHLDA* pleckstrin homology‐like domain family 2, *PI3K/Akt* phosphatidylinositol 3-kinase/Protein Kinase B, *PRP* platelet-rich plasma, *PTEN* phosphatase and tensin homolog deleted on chromosome ten, *RUNX2* RUNX family transcription factor 2, *SDC1* Syndecan 1, *SM-MSCs* synovial membrane MSCs, *TAK1* Transforming Growth Factor-Beta-Activated Kinase 1, *TNFα* tumour necrosis factor α, *UC-MSCs* umbilical cord MSCs, *WNT/β-catenin* Wingless and Int-1/Beta-catenin signalling pathway, *WNT5a/b* Wnt Family Member 5A/5B

## Clinical Trials Using Conditioned Medium

The benefits obtained from using CM in the manipulation of chondrocyte biology and the increasingly well-understood properties of the secretome of MSC-CM may open a new approach to the management of OA [87]. In addition to the current studies, the effects of the CM on the exact large-scale manufacturing process and its composition are needed [29, 134]. Further steps include in vivo studies confirming observed effect CM’s on chondrocytes in in vitro experiments are also necessary [140]. As mentioned above, Chen et al. conducted an in vivo study, but they emphasise that before CM could be used clinically, research on larger animals, animals more alike to humans in size and whose joint movements are more similar to those of humans, is necessary [140]. The final steps that would allow CM to be used in OA therapy are clinical trials, which are underdeveloped and conducted in small numbers.

To date, clinical usage of CM is uncommon. The recently registered trials in the ClinicalTrials.gov database (search performed using the following parameters (Condition or disease: Osteoarthritis; Other terms: conditioned medium OR exosomes OR extracellular vesicles) show eight studies (Table 5) [208]. We have included only six studies in the table because the trials number NCT05546541 and NTC03448796 were only related to the biological profiling of EVs cargo but not their usage as a treatment platform. The newest study NCT05579665 will compare the efficacy of PRP, UC-MSCs-CM and HA in knee osteoarthritis patients. At the time of searching, the trial is active, but yet to recruit. The trial numbered NCT04314661 involved injecting the knee joint using UC-MSC cells or a CM derived from them. On the search date (28 January 2023) the status of the study was ‘recruiting’. The following research, NCT04223622, was designed to investigate the effect of a CM derived from ASCs on human osteochondral explants. The other goal of this study was to compare the effect of CM and extracellular vesicles, and what should support their usefulness as a cell-free therapeutic agent. However, there is a lack of results for this trial due to the ongoing recruitment of the participants. Another exciting study (NCT05060107) is related to the influence of EVs on OA cartilage biology. Researchers are focused on estimating the safety of a single intraarticular injection of EVs derived from MSC in symptomatic mild/moderate knee OA. Due to the lack of ongoing recruitment of the participants, the conclusions are unknown. The study (NCT04225481) involves obtaining an intra-articular stromal vascular fraction (SVF) injection from adipose tissue and then extracting CM from the resulting fraction. The effect of CM will be tested on chondrocytes from patients with OA. Study NCT04223622 is recruiting, while study NCT04225481 has unknown status as the date of analysis. The last searched study (NCT03800810) also has an unknown status. The study was designed to compare the efficacy of UC-MSC implantation, HA therapy and treatment with recombinant human somatropin, which does not directly address the use of a CM. The fewness of clinical trials, undefined protocols and the incomplete understanding of the composition of the CM and its effects argue in favour of developing research towards the understanding and application of CM in the treatment of OA.

**Table 5:** Clinical trials on the usage of CM in the treatment/support of OA registered in the ClinicalTrials.gov database

Study number	Study Title	Summary	Status	Estimated Completion Date
NCT05579665	Effectiveness of PRP, Conditioned Medium UC-MSCs Secretome and Hyaluronic Acid for the Treatment of Knee Osteoarthritis	Comparison of the efficacy of PRP, UC-MSCs-CM or HA injections in the treatment of knee osteoarthritis	Active, not recruiting	May 31, 2023
NCT04314661	Mesenchymal Stem Cell Therapy (MSCs) and Conditioned Medium Therapy for Osteoarthritis (OA)	Comparison of the efficacy of UC-MSCs and secretome between arthroscopy and without arthroscopy intervention in OA patients	Recruiting	December 8, 2024
NCT04223622	Effects of ASC Secretome on Human Osteochondral Explants (ASC-OA)	Investigation of the therapeutic potential of ASC secretome (conditioned medium or extracellular vesicles) on osteochondral explants – a more representative OA model of the physiological situation	Recruiting	December 2022
NCT05060107	Intra-articular Injection of MSC-derived Exosomes in Knee Osteoarthritis (ExoOA-1) (ExoOA-1)	The estimation of safety and outcome after a single injection of exosomes derived from allogenic MSC on the functionality of the patients affected with a moderate stage of advances of knee OA	Not yet recruiting	October 5, 2023
NCT04225481	Evaluation of Enhanced Therapeutic Effect of Adipose Stromal Vascular Fraction Added With Human Platelet Lysate in Treatment of Osteoarthritis	Investigating the effect of adipose tissue-derived-SVF enriched with human platelet lysate and CM isolated from them on OA chondrocytes	Unknown	July 2022
NCT03800810	Implantation of Allogenic Mesenchymal Stem Cell From Umbilical Cord Blood for Osteoarthritis Management	Comparison of the efficacy of UC-MSC in CM, HA and recombinant human somatropin intraarticular injections in patients with OA	Unknown	May 2019

*ASC* adipose stromal cells, *CM* conditioned medium, *HA* hyaluronic acid, *MSC* mesenchymal stromal cells, *OA* osteoarthritis, *PRP* platelet-rich plasma, *SVF* stromal vascular fraction, *UC-MSCs* Umbilical Cord MSCs

## Conclusion

Due to the limitations and invasiveness of traditional methods of OA management, tissue engineering techniques have developed significantly. These methods entail drawbacks, however, associated with the use of cells, such as stromal cells. The beneficial effects exerted by MSCs have been attributed to their paracrine abilities, thanks to the development of stromal cell research. It gave rise to CM, which were previously treated as cell culture biowaste.

The usage of CM in the management of OA has its pros and cons, the advantages, however, outweighing the disadvantages. CM may be obtained from a various stromal cells and iPS cells, but the secretome of the latter is yet to be sufficiently explored. A CM includes proteins, cytokines, growth factors, chemokines and lipids, with both anti-inflammatory and pro-inflammatory effects related to chondrocyte catabolic and anabolic processes.

Since there are several factors included in the CM such as proteins and ncRNA, which have multiple targets in the joint environment, establishing one exact mechanism responsible for the action of secretome derived from MSC is impossible. From the one side, it looks like a continuous interplay between the pro-inflammatory anti-inflammatory factors allows remodelling to occur and ultimately improves cartilage's structure and biomechanical properties. On the other hand, the modification of developing local inflammation is partially the key to the deceleration of OA progression by attenuating activated macrophages and synoviocytes secreting the massive amount of metabolites, harmful for articular cartilage ECM and chondrocytes. As mentioned above the combination of a few protein/ncRNAs inhibit enhanced expression of proteins related to the NF-κB signalling pathway, a master of several inflammation-related processes, and mild modification of the WNT/β-catenin signalling pathway, a key modulator of chondrogenic homeostasis, would provide a potential therapeutic approach in deceleration of OA-related.

The results of most of presented studies on the effects of stromal cell secretome on OA chondrocytes confirmed the beneficial effects of CM, alleviating processes associated with the pathomechanism of the disease.

Further studies leading to standardised protocols for obtaining CMs and EVs, as well as clinical trials to relate the results obtained in in vitro studies to the human body, are needed, however, before CM from MSCs and iPSCs may find application in the treatment of OA.

## Data Availability

Not applicable.

## Abbreviations

ACAN: Aggrecan
ACI: Autologous chondrocyte implantation
ADAMTS: A Disintegrin and Metalloproteinase with Thrombospondin motifs
AD-MSCs: Adipose tissue-derived MSCs
Ang-1 /-2: Angiopoietin-1 /-2
ASCs: Adipose stromal cells
Bax: BCL2 Associated X
B-CECs: Bovine corneal endothelial cells
BM-MSCs: Bone marrow MSCs
CCL2: C-C motif chemokine ligand 2
CDH11: Cadherin 11
circRNA: Circular RNA
CM: Conditioned media
COL10: Collagen type X
COL2A1: Collagen type II α 1 chain
COMP: Cartilage oligomeric matrix protein
CS: Cell sheets
CXCL12: C-X-C motif chemokine 12
CXCR4: C-X-C chemokine receptor type 4
DC: Dendritic cells
DKK-1: Dickkopf 1
DMEM: Dulbecco's Modified Eagle Medium
DMEM/F12: Dulbecco's Modified Eagle Medium/Nutrient Mixture F-12
DPSCs: Dental pulp stromal cells
E2F2: E2F transcription factor 2
ECM: Extracellular matrix
ELF3: E74 Like ETS Transcription Factor 3
ERK: Extracellular-signal-regulated kinase
ERK1/2: Extracellular signal‑regulated protein kinase
ESCs: Embryonic stem cells
EVs: Extracellular vesicles
FBS: Fetal bovine serum
FGF: Fibroblast growth factor
GAGs: Glycosaminoglycans
GIT1: ARF GTPase-Activating Protein
GROα: Growth-regulated α protein
GRPR: Gastrin Releasing Peptide Receptor
HA: Hyaluronic acid
HGF: Hepatocyte growth factor
HIPK3: Homeodomain interacting protein kinase 3
ICAM-1: Intercellular adhesion molecule-1
IFN-γ: Interferon γ
IGF-1: Insulin-like growth factor 1
IL-1Ra: Interleukin-1 receptor antagonist
IL-1β /-6 /-8 /-10: Interleukin 1β /6 /8 /10
iMSCs: induced mesenchymal stromal cells
iPSC: induced pluripotent stem cells
ISCT®: The International Society for Cell and Gene Therapy
K&L: Kellgren and Lawrence
KDMA2: Lysine Demethylase 2A
LIF: leukemia inhibitory factor
lncKLF3-AS1: KLF Transcription Factor 3 Antisense RNA 1
lncRNA: long-non coding RNA
LYRM4: LYR Motif-Containing Protein 4
MACI: matrix-induced autologous chondrocyte implantation
MCP-1: monocyte chemotactic protein 1
METTL3: Methyltransferase 3
MIP1-α: macrophage inflammatory protein 1α
miRNA: micro RNA
MMPs: matrix metalloproteinases
MPCs: mesenchymal chondro-progenitor cells
MSC: mesenchymal stromal cell
MYH9: myosin heavy chain 9
NF-κB: nuclear factor kappa-light-chain-enhancer of activated B cells
NK: natural killer cells
NLRP3: NLR family pyrin domain containing 3
NO: nitric oxide
OA: osteoarthritis
OPG: osteoprotegerin
p21 /p38 /p53: protein 21 /38 /53
PAI: plasminogen activator inhibitor
DDLA-PEG-PDDLA: Poly(D,L-lactide)-block-poly(ethylene glycol)-block-poly(D,L-lactide)
PDGF: platelet derived growth factor
PGE2: prostaglandin-E2
PHLDA2: pleckstrin homology‐like domain family 2
PI3K/Akt: phosphatidylinositol 3-kinase/Protein Kinase B
PRP: platelet-rich plasma
PTEN: phosphatase and tensin homolog deleted on chromosome ten
RA: rheumatoid arthritis
RANKL: receptor activator for nuclear factor κβ ligand
RANTES: regulated upon activation, normal T cell expressed and presumably secreted
ROS: reactive oxygen species
RUNX2: RUNX family transcription factor 2
SA-β-gal: senescence-associated β-galactosidase
SDC1: Syndecan 1
SDF-1: stromal cell-derived factor 1
SEA: N-stearoylethanolamide
SERPINE: serine proteinase inhibitor
SHED: human exfoliated deciduous teeth stromal cells
SM-MSCs: synovial membrane MSCs
SOX5 /6 /9: SRY-Box transcription factor 5 /6 /9
SVF: stromal vascular fraction
TAK1: Transforming Growth Factor-Beta-Activated Kinase 1
TGF-β1/Smad: transforming growth factor β1/suppressor of mothers against decapentaplegic
TIMP-1 /-2: tissue inhibitor of metalloproteinases-1 /-2
TNFα: tumour necrosis factor α
UCB-MSCs: umbilical cord blood MSCs
UC-MSCs: umbilical cord MSCs
VEGF: vascular endothelial growth factor
VIM: vimentin
WJ-MSCs: Wharton's jelly MSCs
WNT/β-catenin: Wingless and Int-1/Beta-catenin signalling pathway
WNT5a/b: Wnt Family Member 5A/5B
